# Hypothesis on the role of cholesterol crystals in spontaneously ruptured aortic plaques: Potential triggers for inflammation and systemic effects

**DOI:** 10.1016/j.ahjo.2025.100507

**Published:** 2025-01-31

**Authors:** Chikao Yutani, Hirotaka Noda, Nobuzo Iwa, Sei Komatsu, Satoru Takahashi, Yoshiharu Higuchi, Kazuhisa Kodama

**Affiliations:** a Division of Pathology, Cardiovascular Center, Osaka Gyoumeikan Hospital, Osaka, Japan; b Non-Profit Organization Japan Vascular Imaging Research Organization, Osaka, Japan; c Department of Medical Technology, Morinomiya University of Medical Sciences, Osaka, Japan; d Division Health Sciences, Area of Medical Laboratory Science and Technology/Department of Clinical Laboratory and Biomedical Sciences, Molecular Pathology, Graduate School of Medicine, Osaka University, Osaka, Japan; e Department of Cardiology, Cardiovascular Center, Osaka Gyoumeikan Hospital, Osaka, Japan; f Cardiovascular Division, Osaka Police Hospital, Osaka, Japan

**Keywords:** Innate immunity, Aorta, Spontaneous ruptured aortic plaque, Cholesterol crystals, Angioscopy

## Abstract

Cholesterol crystals (CCs) are a key component of atherosclerotic plaques and play a pivotal role in plaque progression, rupture, and the resulting inflammatory responses. CCs emboli trigger proinflammatory cytokines which can potentially lead to organ damage. Spontaneously ruptured aortic plaques (SRAPs) are frequently observed via non-obstructive general angioscopy (NOGA) in patients with or suspected coronary artery disease. The release of CCs from SRAPs can activate the innate immune system and induce neutrophil extracellular trap (NET) formation, further exacerbating inflammation. Inflammation levels in SRAPs vary, and the interleukin (IL)-6 ratio may reflect the degree of inflammation. Systemic inflammation induced by CCs may contribute to conditions that may lead to cerebral infarction, and chronic kidney disease. The effects of anti-inflammatory drugs, including IL-6 inhibitors, IL-1β inhibitors, and colchicine, may be evaluated by measuring the IL-6 ratio in SRAPs. This review examined innate immunity mechanisms associated with CCs in SRAPs sampled via NOGA and discussed their systemic impact and potential therapeutic strategies.

## Highlights

• Cholesterol crystals drive inflammation and systemic impact via SRAPs.
• IL-6 ratio quantifies inflammation levels in individual spontaneously ruptured aortic plaques.
• CCs contribute to aging-related organ decline via embolism and immune activation.
• NOGA reveals SRAPs' role in microemboli and chronic systemic inflammation.

## 1 Introduction

Cardiovascular disease is a leading cause of death worldwide [1], with atherosclerosis being a major underlying factor [2]. Atherosclerosis progresses through lipid deposition, causing inflammation, plaque formation, and rupture [3]. Cholesterol crystals (CCs) within plaques have been understudied because of their appearance as ghost images in hematoxylin-eosin (HE) staining. Recent studies have revealed the importance of CCs in plaque progression and rupture [5,6]. Organ damage from CCs, known as cholesterol embolization syndrome (CES), was initially considered primarily an iatrogenic condition following surgeries and catheterization [4]. However, in addition to inflammation, CCs may damage peripheral organs by mechanical obstruction [5,6]. In addition to acute CES, CCs-induced organ damage may also be occurring on a regular daily basis. CCs have been found to circulate, cause organ dysfunction, and contribute to the aging process [7,8]. In this review, we propose a hypothesis that CCs originating from the aorta are potential triggers for inflammation and systemic organ embolism.

## 2 Mechanisms of innate immunity in aortic plaque rupture

**Fig. 1:**
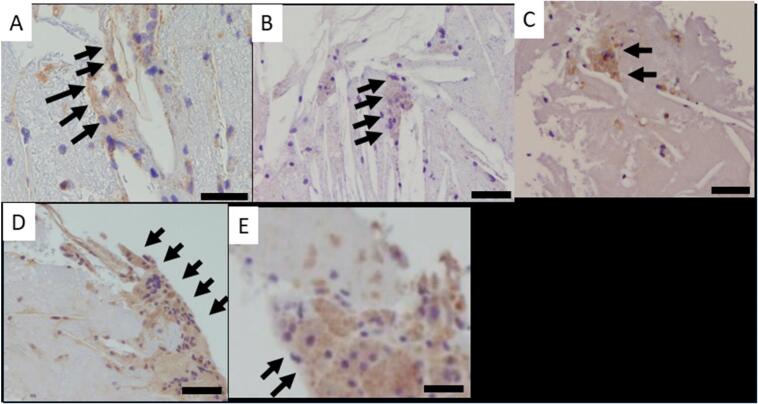
Images of immunostaining of debris from spontaneously ruptured aortic plaques (bar, 20 μm) (A) A representative immunostaining imaging for CD68 showing macrophages. (B) A representative immunostaining imaging for Mincle showing macrophages. Macrophages surrounding empty clefts, thought to be due to CCs, were strongly stained. (C) A representative immunostaining imaging for nucleotidebinding domain, leucine-rich-repeat containing family, pyrin domain-containing 3 (NLRP3). NLRP3 are strongly stained in the macrophages. (D) A representative immunostaining image for interleukin (IL)-1beta. Free macrophages and macrophages surrounding empty clefts were strongly stained. (E) A representative immunostaining imaging for IL-6. IL-6 is stained in the macrophages.

**Fig. 2:**
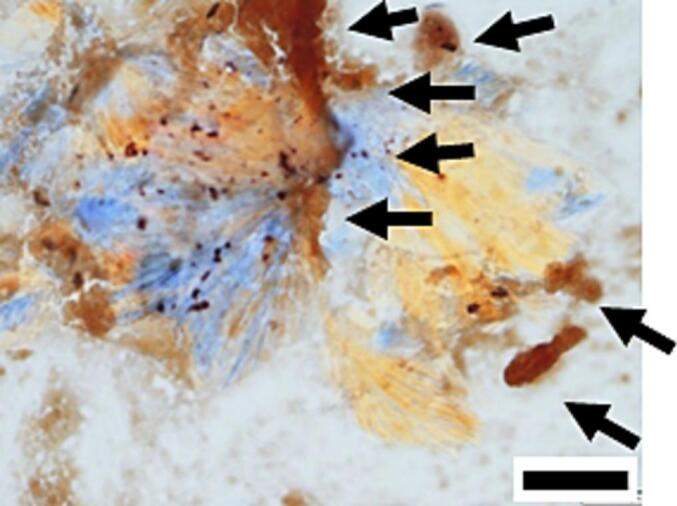
A representative polarized light microscopic images of Vital Sternheim-Malbin stain. For debris from spontaneously ruptured aortic plaques (bar, 20 μm). Macrophages recognized and engulfed CCs.

In the early stages of aortic atherosclerosis, macrophages phagocytose lipoproteins and transform into foam cells, transforming into M1 macrophages [9]. These foam cells accumulate and expand to form fatty streaks [10]. M1 macrophages maintain inflammatory responses by secreting proinflammatory cytokines and reactive oxygen species [11], while interacting with vascular smooth muscle cells to enhance intimal thickening [12,13]. As endothelial cell permeability increases, macrophages penetrate plaques [14]. Persistent inflammation drives macrophage apoptosis, resulting in a necrotic core in the absence of efficient efferocytosis [15]. The innate immune system recognizes pathogens through pattern-recognition receptors [16]. Macrophage activation, NLRP3 inflammasome, and macrophage-inducible C-type lectin (MINCLE} may contribute to plaque rupture (Fig. 1) [6,14,17]. Studies have shown macrophages adhering to CCs in SRAPs [18], indicating MINCLE-CC binding (Fig. 2) [19].

**Fig. 3:**
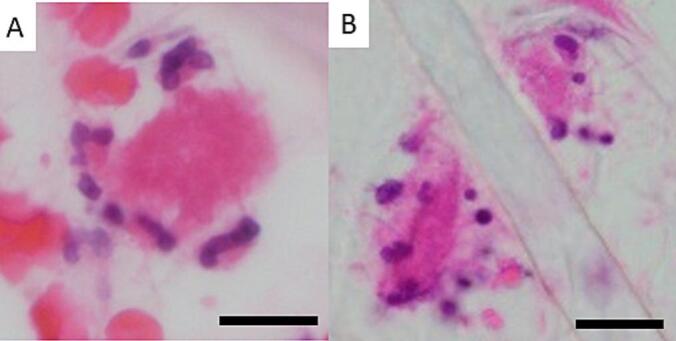
Neutrophils in contact with fibrin. (bar, 20 μm) A villous and reticular NET formation can be observed from neutrophils. (A). The neutrophil's segmentation has disappeared, and the nucleus has formed a single mass, losing function and about to undergo netosis. (B). It is releasing clusters of histones in a mesh-like pattern.

**Fig. 4:**
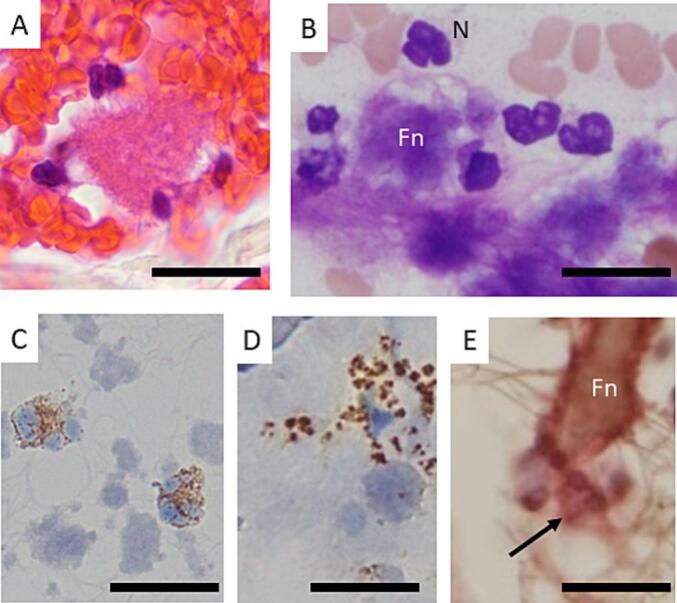
Nets Formation in SRAPs. (bar, 20 μm) (A): Nets formation, (B): PTAH staining showing fibrin (F) and neutrophils (N), (C, D): MPO myeloperoxidase granules (MPO staining), MPO granules within the nucleus attempting to move outside the cell (C). MPO granules that have exited the cell (D). (E): Immunostaining for neutrophil elastase (red) and fibrinogen (Fn, brown). Neutrophil elastase adheres around fibrinogen. (For interpretation of the references to colour in this figure legend, the reader is referred to the web version of this article.)

NLRP3 activates neutrophils and supports NET formation [20], observed in SRAP samples (Fig. 3, Fig. 4). These mechanisms might trigger NET expression, complementing conventional rupture mechanisms [6]. Mass spectrometry revealed docosahexaenoic acid in CC metabolism [18], while blood samples showed inflammatory proteins persisting post-rupture, suggesting inflammation-related cytokines maintain fatty acid metabolism before plaque rupture [21,22].

## 3 Non-obstructive general angioscopy system: insights into aortic plaques and CCs

**Fig. 5:**
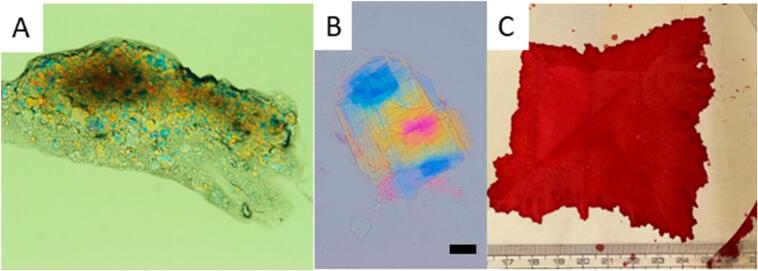
Sampling of spontaneous rupture aortic plaques (SRAPs) (A) Polarized light microscopic images of debris. (B) Free multi-layered cholesterol crystals. (bar, 20 μm) (C) Macroscopic image of SRAPs on a filter paper.

Aortic plaques have traditionally been evaluated using non-invasive imaging techniques like CTA, MRI, and transesophageal echocardiography [23]. The non-obstructive general angioscopy (NOGA) system employs a fiber-optic catheter to precisely visualize and identify plaques and vascular damage within the aorta and peripheral arteries [7,8,23]. The system has shown effectiveness in detecting spontaneously ruptured aortic plaques (SRAPs) in cases where computed tomography angiography (CTA) fails to identify them [23]. NOGA enables in situ sampling of components from SRAPs [7,17]. CCs in SRAPs can be detected using polarizing microscopy of blood samples or through filter paper examination with HE staining [7,24]. One method uses a polarizing microscope to examine 10 mL of blood or debris obtained during aortic plaque rupture (Fig. 5A and B). Another method involves spreading the sample onto filter paper (Fig. 5C), fixing the filter paper in formalin, creating paraffin sections including the filter paper, staining with HE, and examining it under an optical microscope. According to NOGA studies, SRAPs occurred in 80.9 % of cases with or suspected coronary artery disease [7], with frequency increasing with age. Scattering-type plaques, puff ruptures and puff-chandelier plaques are commonly associated with CCs [7,25]. The detection rate of CCs using polarized light microscopy with HE staining is approximately twice that of HE staining alone [25]. This improved detection rate is primarily because free CCs, not contained within atheroma components, dissolve during the organic solvent preparation for HE staining [7,24,26]. In puff-chandelier ruptures, the median number of CCs is 12,727, while the number of puff ruptures is 3182 per 10 mL of sampled blood [25]. These findings indicate that CCs are continuously being released downstream. However, studies using NOGA suggest that plaque rupture is a continuous process involving CC emboli release. Studies indicate plaque rupture is a constant process rather than a single event, and cases have shown coronary plaque rupture persisting for 1 month [27] and aortic plaque rupture for 2 years [28]. While CCs are detected in 9 % of femoral artery cases [29], their presence has been noted in blood samples taken after the appearance of CES symptoms [30]. SRAPs, especially scattering-type plaques, are frequently associated with CES [31], indicating the potential for continuous release of CCs from SRAPs.

## 4 Comparison of inflammation levels of CCs within SRAPs

CCs exist in monolayer and multilayer forms, varying in size [7]. They can damage blood vessels and capillaries [7,22], causing mechanical ischemia due to their sharp morphology [5,6] while activating innate immunity [17,32,33]. In SRAPs, macrophages recognize and interact with CCs, triggering the NLRP3 inflammasome and inflammatory cytokines [17,18,32,34]. Immunostaining showed positive CD68, NLRP3, IL-1b, and IL-6 results in most samples [17], confirming innate inflammation within plaques.

Inflammatory blood markers may reflect systemic inflammation against unknown foreign substances, and may also be elevated due to the inflammation produced by the atherosclerotic plaque [4,35]. To evaluate plaque-derived inflammation, the IL-6 level at the aortic cusp was used as a baseline, and the IL-6 level for each SRAP was divided by this baseline, defining the result as the IL-6 ratio [29]. Sampling flowing blood has limitations; however, the IL-6 ratio minimizes the influence of baseline inflammation levels. The median IL-6 ratio in all sampled SRAPs was 1.10 [29]. Consequently, puff-chandelier ruptures containing numerous CCs exhibited significantly higher IL-6 ratios than puff ruptures containing fewer CCs. A moderate correlation was observed between the number of CCs and the IL-6 ratio, although no correlation was found between the number of CCs and IL-6 values⁠ [29].

The results suggest a positive correlation between CC quantity and inflammation, as adjusted by the IL-6 ratio. In other words, CCs likely initiate pathways leading to IL-6 production⁠ [29]. The IL-6 ratio may effectively track inflammation changes in individual plaques⁠. These findings highlight the variation among SRAPs and the significance of identifying individual SRAPs' inflammation levels and progression.

It is also hypothesized that inflammasome components and inflammatory cytokines are spread through peripheral arteries and arterioles following plaque rupture. The IL-6 ratios in the brachial and femoral arteries are 1.06 and 1.11, respectively [29]. The decline in inflammation is not clearly understood; it may be due to dilution in aortic blood or the recirculation of CCs to the liver via high-density lipoprotein (HDL) for hormone synthesis. Furthermore, peripheral inflammation may result from peripheral arterial and arteriole damage and the effects of ongoing inflammatory cytokines at different times.

## 5 Impact and treatment of SRAPs

Registry studies have shown links between aortic plaques and systemic embolic events [36]. Studies have found connections between proximal aortic arch plaques and brain white matter lesions. SRAPs from the ascending aorta to the proximal aortic arch are particularly implicated in cerebral infarctions where other causes are ruled out [[37], [38], [39], [40]]. SRAPs from the ascending aorta to the proximal aortic arch are implicated in cerebral infarctions in patients without atrial fibrillation or significant abnormalities in the carotid or cerebral arteries⁠. This suggests that the inflammasomes from embolic sources⁠ and the inflammasomes from locally derived plaques in the brain should be considered [41,42]. We previously reported a case of lower extremity arterial disease with recurrent occlusion due to an aortic embolic source [42]. In a case report, CCs were found obstructing the blood flow in a setting of critical limb ischemia, which resulted in an amputation. Numerous CCs were detected at the amputation cross-section [31]. The embolic source of SRAPs was determined by comparing the embolic source's similarities with the embolus plaque's components in composition, fat globules, and cavities in HE stains [42]. Chronic, continuous scattering of CCs may play a role in gradual declines in abilities associated with aging, such as chronic kidney disease and dementia, though this remains challenging to substantiate [7,8,23,[43], [44], [45], [46]].

Given the temporal continuity of SRAPs, evaluating each SRAP and its degree of inflammation is a new approach for ⁠assessing anti-inflammatory drug efficacy. While IL-6 and IL-1β inhibitors show promise [47,48], colchicine has emerged as a cost-effective alternative [49,50]. Colchicine significantly reduced cardiovascular event recurrence in high-risk patients, suggesting mechanisms beyond anti-inflammatory effects⁠. Interestingly, research has shown that colchicine significantly impacts CCs [51]. Experimental studies indicate colchicine reduces cardiovascular events in high-risk patients and affects CC formation and breakdown [52]. However, further research is needed to understand its long-term effects.

## 6 Conclusion

Despite the continuous release of extensive CCs into arterial blood from SRAPS, detection of immediate symptomatic organ infarction remains rare. The body likely mobilizes macrophages, endothelial cells, and neutrophils to engulf and digest CCs, preventing infarction. However, the fate of small, unrecognized, or trapped CCs is still being determined. Two possibilities are proposed: recycling of CCs and asymptomatic organ damage. Dissolved CCs may return to the liver via HDL and re-enter the cholesterol cycle for hormone synthesis [51,53]. In contrast, CCs may embolize various organs, potentially creating ischemic lesions over time. This process could underline organ decline, which is traditionally attributed to aging. Atherosclerosis understanding has evolved from historical theories to modern concepts of immunothrombosis, potentially leading to improved treatments.

## Ethical

This manuscript has not been published or presented elsewhere in part or in entirety and is not under consideration by another journal. We have read and understood your journal's policies, and we believe that neither the manuscript nor the study violates any of these.

This study was conducted in compliance with the 1975 Declaration of Helsinki and was approved by the local ethics committee of Osaka Gyoumeikan Hospital (21-0003), and all patients provided written informed consent.

## CRediT authorship contribution statement

**Chikao Yutani:** Writing – original draft, Methodology. **Hirotaka Noda:** Writing – review & editing, Methodology, Investigation, Data curation. **Nobuzo Iwa:** Writing – review & editing, Investigation. **Sei Komatsu:** Writing – original draft, Methodology. **Satoru Takahashi:** Investigation, Data curation. **Yoshiharu Higuchi:** Writing – original draft, Methodology. **Kazuhisa Kodama:** Validation, Project administration, Conceptualization.

## Funding

None.

## Declaration of competing interest

Sei Komatsu is a consultant of Nemoto Kyorin-Do. Co., ltd. Other authors declare that they have no known competing financial interests or personal relationships that could have appeared to influence the work reported in this paper.
